# Novel Polymorphisms and Genetic Features of the Prion Protein Gene (*PRNP*) in Cats, Hosts of Feline Spongiform Encephalopathy

**DOI:** 10.3390/genes12010013

**Published:** 2020-12-24

**Authors:** Hyeon-Ho Kim, Yong-Chan Kim, Kiwon Kim, An-Dang Kim, Byung-Hoon Jeong

**Affiliations:** 1 Korea Zoonosis Research Institute, Jeonbuk National University, Iksan, Jeonbuk 54531, Korea; khh7051@jbnu.ac.kr (H.-H.K.); kych@jbnu.ac.kr (Y.-C.K.); 2 Department of Bioactive Material Sciences and Institute for Molecular Biology and Genetics, Jeonbuk National University, Jeonju, Jeonbuk 54896, Korea; 3 Haemalken Animal Hospital, Yangju, Gyeonggi 11492, Korea; kkw0075@hanmail.net; 4 Cool-Pet Animal Hospital, Anyang, Gyeonggi 14066, Korea; Kad7582@hanmail.net

**Keywords:** feline spongiform encephalopathy, FSE, prion diseases, cat, dog, prion protein gene (*PRNP*), single nucleotide polymorphism (SNP)

## Abstract

Prion diseases are fatal neurodegenerative disorders characterized by vacuolation and gliosis in the brain. Prion diseases have been reported in several mammals, and genetic polymorphisms of the prion protein gene (*PRNP*) play an essential role in the vulnerability of prion diseases. However, to date, investigations of *PRNP* polymorphisms are rare in cats, which are the major host of feline spongiform encephalopathy (FSE). Thus, we investigated the genetic polymorphisms of the cat *PRNP* gene and analyzed the structural characteristics of the PrP of cats compared to those of dog, prion disease-resistant animal. To investigate the genetic variations of the cat *PRNP* gene in 208 cats, we performed amplicon sequencing and examined the genotype, allele and haplotype frequencies of cat *PRNP* polymorphisms. We evaluated the influence of cat *PRNP* polymorphisms using PolyPhen-2, PANTHER, PROVEAN and AMYCO. In addition, we carried out structural analysis of cat PrP according to the allele of nonsynonymous single nucleotide polymorphism (SNP) (c.457G > A, Glu153Lys) using Swiss-PdbViewer. Finally, we compared the structural differences between cat and canine PrPs for SNPs associated with prion disease resistance in dogs. We identified a total of 15 polymorphisms, including 14 novel SNPs and one insertion/deletion polymorphism (InDel). Among them, Glu153Lys was predicted to affect the structural stability and amyloid propensity of cat PrP. In addition, asparagine at codon 166 of cat PrP was predicted to have longer hydrogen bond than aspartic acid at codon 163 of canine PrP. Furthermore, substitution to dog-specific amino acids in cat PrP showed an increase in structural stability. To the best of our knowledge, this is the first study regarding the structural characteristics of cat *PRNP* gene.

## 1. Introduction

Prion diseases, also called transmissible spongiform encephalopathies (TSEs) are lethal neurodegenerative diseases caused by the accumulation of abnormal prion protein (PrP^Sc^) [1]. PrP^Sc^ is originates from the misfolding of normal prion protein (PrP^C^), however, the exact mechanisms of the conversion process are still unknown [2]. Prion diseases occur in several mammals, including Creutzfeldt-Jakob disease (CJD) in humans, bovine spongiform encephalopathy (BSE) in cattle, scrapie in sheep and goats, chronic wasting disease (CWD) in deer and elk, transmissible mink encephalopathy (TME) in mink and feline spongiform encephalopathy (FSE) in Felidae [3,4,5,6,7,8,9,10,11,12,13,14,15,16].

FSE occurred with similar temporal and geographical distributions as the BSE outbreak. The first FSE case was reported in domestic cats in 1990 [17]. From 1990 to 2007, FSE was reported in various feline animals, including domestic cats, cheetahs, lions, pumas, tigers, ocelots, Asian leopard cats and Asian golden cats [5,6,8,18,19,20] in the United Kingdom (UK), other European countries and Australia [21,22,23,24,25]. In addition, since FSE has shown immunohistochemical and biochemical characteristics similar to those of BSE, it is assumed that the origin of FSE is BSE [23,26]. However, although cats and dogs showed similar habitats, eating habits and PrP sequences, dogs showed the opposite results in prion infection and transmission compared to cats [27,28,29,30,31]. During the outbreak of BSE in the UK, prion disease was not reported in dogs. In previous studies, dogs have shown resistance to several types of prion diseases [29,32,33]. Madin Darby canine kidney (MDCK) cells showed resistance to prion disease agents, including sporadic CJD and Rocky Mountain Laboratory (RML) scrapie strains [32]. The canine PrP transgenic mouse has shown resistance to prion strains, including SSBP/1, BSE-C, CWD, sheep-BSE, cat CWD, BSE-L and atypical scrapie [29]. Compared to cat and rabbit PrPs, canine PrP showed resistance to conversion to PrP^Sc^ by protein misfolding cyclic amplification (PMCA) [33]. Thus, comparative analysis of PrP between cats and dogs will be an essential basic study to reveal genetic factors of prion disease.

The susceptibility of prion disease is associated with polymorphisms of the prion gene family [34]. Among them, the prion protein gene (*PRNP*) encodes PrP, and its polymorphism has been reported to be associated with susceptibility to prion disease in various prion disease-susceptible animals, including humans, sheep, goats and cattle [35,36,37,38,39,40,41,42,43]. However, aspartic acid at codon 163 of canine PrP is a dog-specific amino acid and plays a pivotal role in the resistance of prion disease [29,44]. This amino acid contributes to the resistance of prion disease through the formation of longer α-helix 1 and the disappearance of the β-sheet structure compared with asparagine at codon 159 of hamster PrP [44]. In addition, transgenic mice expressing Asn158Asp mouse PrP showed resistance to infection with several prion agents including RML, 301C and 22L [45]. On the other hand, transgenic mice expressing Asp163Asn canine PrP revealed susceptibility to prion strains, including sheep-BSE [29]. In recent studies, the nonsynonymous SNPs have been reported in dogs [28,31,46]. Among these SNPs, the Asp163Glu SNP was predicted to have no impact on canine PrP structure [31]. However, the Asp182Glu and Asp182Gly SNPs were predicted to affect the stability of canine PrP by changing the number of hydrogen bonds [31].

To date, although cats are hosts of FSE, studies on polymorphisms of the cat *PRNP* gene are rare [28]. In addition, there is no report on polymorphisms of the cat *PRNP* gene in FSE-infected animals. Since the genetic polymorphisms of the cat *PRNP* gene can be associated with susceptibility to prion disease, identification and functional analysis of polymorphisms of the *PRNP* gene in large samples of healthy cats are an essential baseline study to identify potential FSE-related genetic factor. In addition, since dog is a unique prion disease-resistant animal in Carnivora, the comparative studies, including structural characterization between cat and canine PrPs, may contribute to understanding the pathogenesis mechanism of prion disease.

In the present study, we performed amplicon sequencing in the cat *PRNP* gene and investigated the genotype, allele and haplotype frequencies of the cat *PRNP* polymorphisms in 208 cats. In addition, we performed a linkage disequilibrium (LD) test among *PRNP* polymorphisms. We also performed *in silico* and 3D structure analyses to identify the influence of polymorphisms in the cat *PRNP* gene using PolyPhen-2, PANTHER, PROVEAN, AMYCO and Swiss-Pdbviewer. Furthermore, we compared the difference in the hydrogen bonds of amino acids associated with resistance to prion disease between cat and canine PrPs. Finally, we investigated the impact of the substitution aspartic acid at codon 166 on cat PrP to dog-specific amino acids

## 2. Materials and Methods

### 2.1. Ethical Statement

Animal experimental procedures were approved by the Institutional of Animal Care and Use Committee (IACUC) of Jeonbuk National University (IACUC number: CBNU 2019-00077). All experiments with domestic cats were followed by the Korea Experimental Animal Protection Act.

### 2.2. Samples

A total of 208 cat samples, including 119 tissues and 89 whole blood samples, were provided from Cool-Pet and Haemalgeun animal hospitals in the Republic of Korea. Blood samples were collected using ethylenediaminetetraacetic acid (EDTA). These samples are byproducts of castration and health medical examinations performed by veterinary surgeons. The tissue samples consisted of testes and ovaries of 2 cat breeds (1 Persian and 118 Korean Domestic Shorthair). Whole blood samples of 89 cats consist of 18 cat breeds (50 Korean Domestic Shorthair, 9 Persian, 4 Siamese, 3 Norwegian Forest, 3 Scottish Fold, 3 Turkish Angora, 2 Abyssinian, 2 Bengal, 2 British Shorthair, 2 Sphynx, 2 Russian Blue, 1 American Curl, 1 American Shorthair, 1 Malaysian Domestic Shorthair, 1 Minuet (another name, Napoleon), 1 Munchkin, 1 Ragdoll and 1 Siberian).

### 2.3. Genetic Analysis

Genomic DNA extraction was performed from tissue samples and whole blood samples. In the tissue samples, genomic DNA extraction was performed using Labopass Tissue Genomic DNA Isolation Kit Mini (Cosmogenetech, Seoul, Korea). In the blood samples, genomic DNA extraction was performed using the Bead Genomic DNA Prep Kit (Biofact, Daejeon, Korea). Polymerase chain reaction (PCR) was performed with a specific primer set for the cat *PRNP* gene (forward: 5′-CCGAGTGGTTCCAACATGAA-3′, reverse: 5′-CTAAAGGGCTGTAGGTAGACAC-3′). These primers were designed based on the DNA sequence of the cat *PRNP* gene (GenBank ID: EU341499.1) to amplify the open reading frame (ORF) of the cat *PRNP* gene. The PCR mixture was composed of 2.5 µL of 10× *Taq* polymerase reaction buffer (25 mM MgCl_2_ mixed), 5 µL of 5× band helper, 1 µL of dNTP mixture (each 10 mM), 1 µL of each primer (10 µM), 0.2 µL of *Taq* DNA polymerase (5 U/µL, Biofact, Daejeon, Korea) and nuclease-free water to a total volume of 25 µL. The PCR experimental conditions were as follows: 1 cycle of predenaturation at 95 °C for 2 min, 34 cycles of denaturation at 95 °C for 20 sec, annealing at 60 °C for 40 sec, elongation at 72 °C for 1 min and 1 cycle of final elongation at 72 °C for 5 min. PCR products were purified using a FavorPrepGel/PCR Purification Mini Kit (FAVORGEN, Pingtung County, Taiwan). Purified PCR products were sequenced by using an ABI 3730 Capillary Sequencer (ABI, Foster city, CA, USA). The sequencing data were analyzed by Finch TV (Geospiza Inc., Seattle, WA, USA).

### 2.4. Statistical Analyses

Hardy-Weinberg equilibrium (HWE), LD and haplotype analyses of cat *PRNP* polymorphisms were performed using Haploview Version 4.2 (Broad Institute, Cambridge, MA, USA). The LD score was measured with r^2^ value.

### 2.5. Sequence Alignment

The sequence alignment of cat and canine PrP was performed by Clustal Omega. Information on the canine PrP sequence (ACO71291.1) was obtained from GenBank at the National Center for Biotechnology Information (NCBI).

### 2.6. In Silico Analysis

We used the PROVEAN, PolyPhen-2 and PANTHER programs to evaluate the influence of nonsynonymous SNPs and insertion/deletion polymorphism (InDel) on cat PrP and AMYCO to evaluate alterations in the amyloid propensity of cat PrP according to alleles of nonsynonymous SNPs and InDel. PolyPhen-2 predicts the effect of amino acid substitution according to the position-specific, independent count (PSIC) score and indicates 3 types of functional changes: “benign”, “probably damaging” and “possibly damaging”. PANTHER uses a hidden Markov model (HMM) based on a statistical modeling method to evaluate scores according to amino acid substitution. PANTHER predicts amino acid substitutions into 3 types: “probably damaging” (450 my < score), “possibly damaging” (200 my < score < 450 my) and “probably benign” (score < 200 my). PROVEAN predicts the influence on amino acid variations of protein with a score below −2.5 being “deleterious” and a score above −2.5 being “neutral”. The predictions including “benign”, “probably benign”, “probably damaging” and “possibly damaging” by PolyPhen-2, PANTHER and PROVEAN indicate a degree of structural and functional changes of template protein according to the substitution of amino acid. AMYCO uses the combination of the PAPA score and pWALTZ score to evaluate protein amyloid propensity. An AMYCO score below 0.45 indicates low amyloid propensity, and an AMYCO socre above 0.74 indicates high amyloid propensity.

### 2.7. 3D Structure Analysis in the Cat and Canine PrPs

Information on the cat PrP sequence was obtained from this study and the 3D structure of cat PrP was predicted by the SWISS-MODEL program. Information on the 3D structure of canine PrP (1XYK) was obtained from the Protein Data Bank (PDB). The structural change according to nonsynonymous SNPs found in this study was determined using the Pdb-Viewer program to analyze the 3D structure. We performed hydrogen bond analysis and the threshold of the hydrogen bond distance was 2.35 to 3.2 Å.

## 3. Results

### 3.1. Investigation of Polymorphisms of the PRNP Gene in 208 Cats

**Figure 1:**
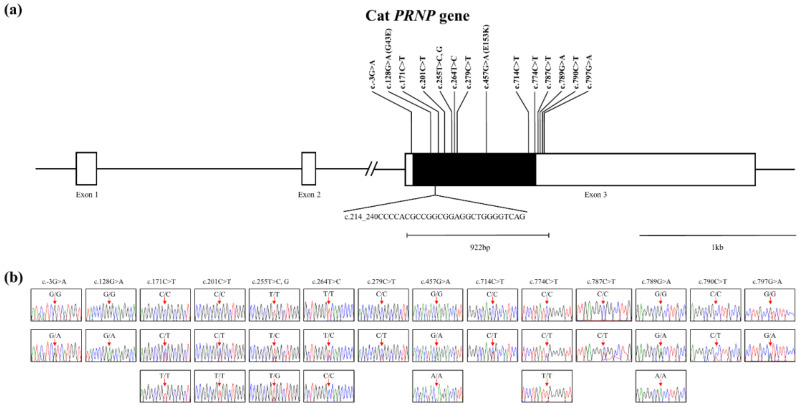
Novel polymorphisms of the cat prion protein gene (*PRNP*) identified in this study. (**a**) Gene map and polymorphisms identified in the cat *PRNP* gene. The open reading frame (ORF) within exon 3 is indicated by a shaded black block, and the 5′ and 3′ untranslated regions (UTRs) are indicated by white blocks. Horizontal bars with edges indicate the regions sequenced. The vertical line and folded line indicate the single nucleotide polymorphisms (SNPs) found in this study. The Y-shaped bar indicates the nonapeptide deletion polymorphisms identified in the cat *PRNP* gene. (**b**) Electropherogram of 14 novel SNPs: c.-3G>A in the 5′ UTR. c.128G>A, c.171C>T, c.201C>T, c.255T>C, G, c.264T>C, c.279C>T, c.457G>A, c.714C>T and c.774C>T in the ORF. c.787C>T, c.789G>A, c.790C>T and c.797G>A in the 3’ UTR. Four colors indicate individual bases of DNA sequence using an ABI 3730 automatic sequencer (blue: cytosine, red: thymine, black: guanine, green: adenine).

**Figure 2:**
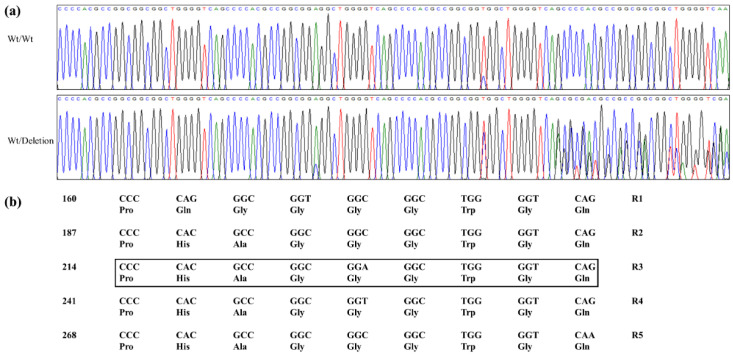
Nonapeptide deletion polymorphism of the cat prion protein gene (*PRNP)* gene identified in this study. (**a**) Electropherograms showing the polymorphism at the nonapeptide repeat region. The upper panel indicates insertion/insertion homozygosity of the cat *PRNP*. The lower panel indicates InDel heterozygosity of the cat *PRNP*. (**b**) The nucleotide and amino acid sequences of the nonapeptide repeat region of the cat *PRNP* gene. The black box indicates the location of nonapeptide InDel of the cat *PRNP* gene (c.214_240CCCCACGCCGGCGGAGGCTGGGGTCAG; p.72_80delPHAGGWGQ).

We performed genotyping with amplicon sequencing data of the *PRNP* gene in cats and identified 14 novel SNPs: c.-3G>A in the 5′ untranslated (UTR) region and c.128G>A, c.171C>T, c.201C>T, c.255T>C, G, c.264T>C, c.279C>T, c.457G>A, c.714C>T, c.774C>T in the ORF region and c.787C>T, c.789G>A, c.790C>T and c.797G>A in the 3′ UTR region (Figure 1). Among the 14 SNPs, c.128G>A (Gly43Glu) and c.457G>A (Glu153Lys) are nonsynonymous SNPs (Figure 1a). We also identified an InDel, c.214_240delCCCCACGCCGGCGGAGGCTGGGGTCAG (p.72_80delPHAGGGWGQ) in the tandem repeat region (Figure 1a and Figure 2a,b). This InDel was located in the nonapeptide repeat R3 region (Figure 2). The genotype and allele frequencies of the 15 polymorphisms are described in Table 1. We also carried out LD analysis among 15 polymorphisms of the cat *PRNP* gene by calculating the r^2^ values (Table 2). Among them, the SNPs c.-3G>A and c.171C>T showed strong LD (r^2^ > 0.3) with c.797G>A and c.201C>T, respectively. Next, we performed haplotype analysis of 15 polymorphisms of the cat *PRNP* gene and found seven major haplotypes of cat *PRNP* polymorphisms (Table 3). Among them, four major haplotypes were observed at frequencies greater than 10%: GGCTWtTTCGCCCGCG (29.8%), GGTCWtTTCGCCCGCG (22.0%), GGTCWtTTCGCTCGCG (11.4%) and GGCCWtTCCGCCCGCG (10.1%).

### 3.2. Investigation of the Influence on Nonsynonymous Polymorphisms of Cat PrP

We estimated the influence on polymorphisms of cat PrP using PolyPhen-2, PANTHER and PROVEAN (Table 4). The nonsynonymous SNP, c.128G>A (Gly43Glu) was estimated to be “probably damaging” with a score of 1.0 by PolyPhen-2. However, this SNP was estimated to be “neutral” with a score of -1.381 by PROVEAN (Table 4). The InDel, c.214_240delCCCCACGCCGGCGGAGGCTGGGGTCAG was estimated to be “deleterious” with a score of −13.052 by PROVEAN. The SNP, c.457G>A (Glu153Lys) was estimated to be “probably damaging” and “possibly damaging” with scores of 0.998 and 361 by PolyPhen-2 and PANTHER, respectively. In contrast, this SNP was estimated to be “neutral” with a score of −1.691 by PROVEAN.

### 3.3. Evaluation of Amyloid Propensity of Cat PrP according to of Polymorphism Alleles

**Figure 3:**
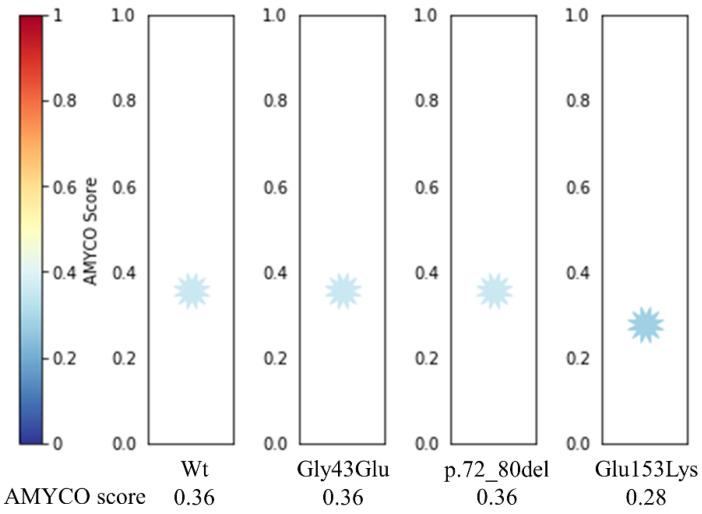
Prediction of amyloid propensity of cat prion protein (PrP) according to alleles of polymorphisms using AMYCO. Blue and red colors in AMYCO score indicate low and high amyloid propensities of the protein, respectively.

We investigated the amyloid propensity of cat PrP according to alleles of nonsynonymous SNPs and InDel (Figure 3). The scores of the c.128G>A (Gly43Glu) SNP and the InDel were equal to that of Wt (0.36) by AMYCO. However, the score of the c.457G>A (Glu153Lys) SNP was lower (0.28) than that of Wt.

### 3.4. Impact of Nonsynonymous SNPs on the 3D Structure of Cat PrP

**Figure 4:**
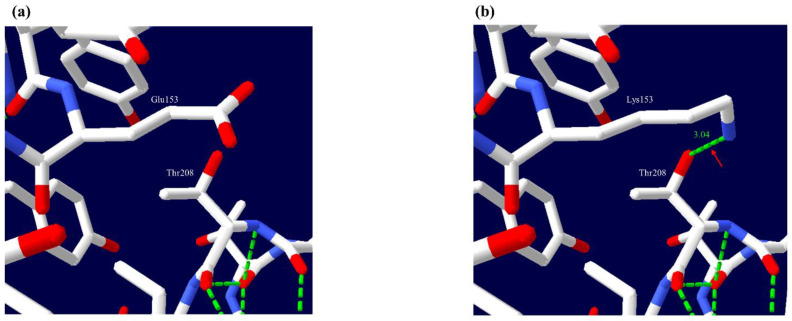
3D structural analysis of cat prion protein (PrP) according to alleles of Glu153Lys. (**a**) 3D structure of cat PrP with Glu153 allele. (**b**) 3D structure of cat PrP with Lys153 allele. Green dotted lines indicate hydrogen bonds. The red arrow indicates the hydrogen bond between Lys153 and Thr208 of cat PrP.

To identify the influence of nonsynonymous SNP on cat PrPs, we performed 3D structure analysis using Swiss-PdbViewer (Figure 4). The amino acid sequence of the previously reported 3D structure of the cat PrP (1XYJ) was different from that of cat PrP found in the study. Thus, based on the amino acid sequence of cat PrP found in this study, we performed homology-based modeling using SWISS-MODEL and analyzed the hydrogen bonds of cat PrP according to alleles of the nonsynonymous SNP. There was a difference in the number of hydrogen bonds between cat PrP with the Glu153 allele and that with the Lys153 allele. In detail, Glu153 was predicted to have no hydrogen bond with other amino acids (Figure 4a), and Lys153 was predicted to have a hydrogen bond (3.04 Å) with Thr208 (Figure 4b).

### 3.5. Comparison of Tandem Repeat Regions of PrP in Several Mammals

**Figure 5:**
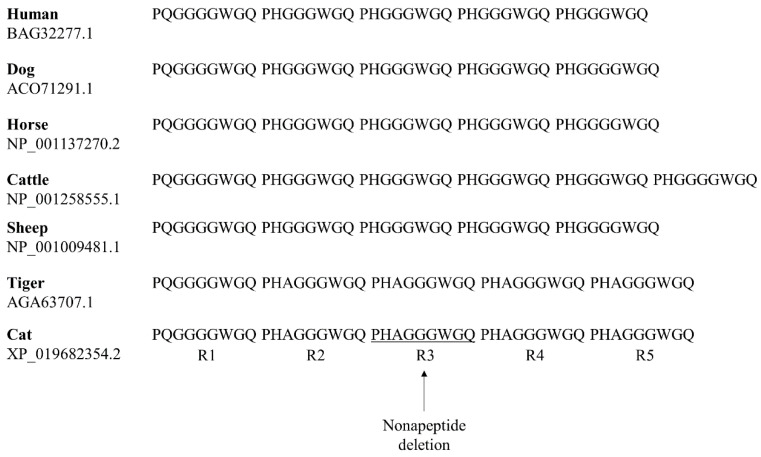
Comparisons of tandem repeat sequences of prion protein (PrP) in humans, dogs, horses, cattle, sheep, tigers and cats. Tandem repeat sequences were obtained from GenBank at the National Center for Biotechnology Information (NCBI), including human (*Homo sapiens*, BAG32277.1), dog (*Canis lupus familuarus*, ACO71291.1), horse (*Equus caballus*, NP_001137270.2), cattle (*Bos taurus*, NP_001258555.1), sheep (*Ovis aries*, NP_001009481.1), tiger (*Panthera tigris*, AGA63707.1) and cat (*Felis catus*, XP_019682354.2). The underline indicates the deletion polymorphism found in this study.

The tandem repeat regions of feline species, including cats and tigers, are different from those of other mammals (Figure 5). The tandem repeats of mammalians including humans, are octapeptide repeat; however, those of feline species, including cats, are nonapeptide repeats. The R1 repeat in the tandem repeat region is PQGGGGWGQ in humans, dogs, horses, cattle, sheep, tigers and cats. The tandem repeat region of the PrP sequence in the feline species has an additional alanine, similar to PHAGGGWGQ.

### 3.6. A Structural Comparison of PrP between Asp163 in Dogs and Asn166 in Cats

**Figure 6:**
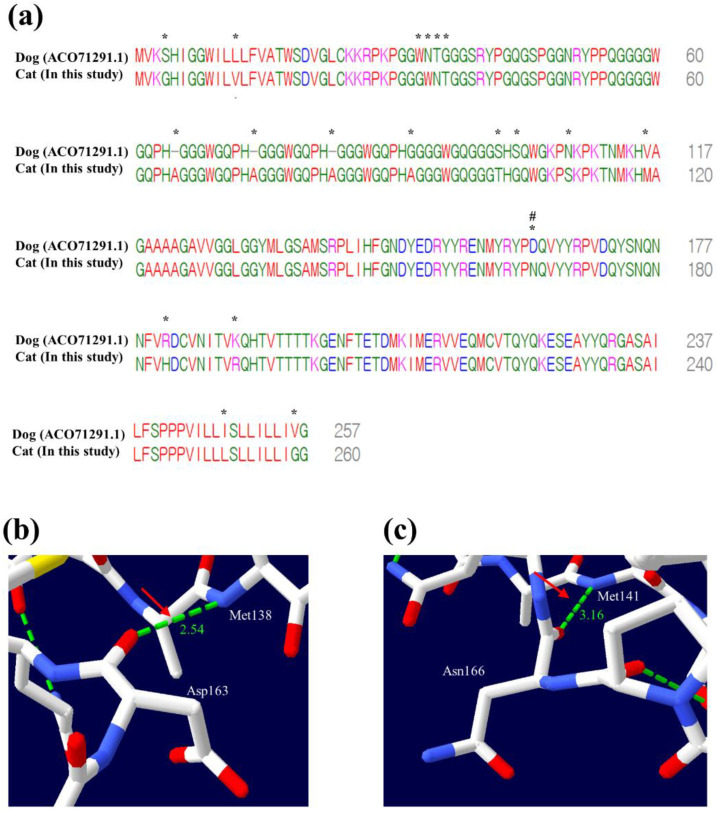
Comparison of amino acid and 3D structures between cat and canine prion proteins (PrPs). (**a**) Comparison of amino acid sequences of PrP between cats and dogs. The cat PrP sequence was obtained from this study. The canine PrP sequence (*Canis lupus familiaris*, ACO71291.1) was obtained from GenBank at the National Center for Biotechnology Information (NCBI). Asterisks indicate the different amino acids of PrP between cats and dogs. Sharp (**#**) indicates an amino acid associated with the resistance of prion disease in canine PrP. (**b**) 3D analysis of the Asp163 of canine PrP. Green dotted lines indicate hydrogen bonds. The red arrow indicates the hydrogen bond between Asp163 and Met138. (**c**) 3D analysis of the Asn166 of cat PrP. Green dotted lines indicate hydrogen bonds. The red arrow indicates the hydrogen bond between Asn166 and Met141.

In previous studies, Asp163 in canine PrP was associated with prion disease resistance [31]. Thus, we performed sequence alignment between cat and canine PrPs (Figure 6a). In cat PrP, Asn166 is homologous to Asp163 of canine PrP (Figure 6a). We performed 3D structural analysis of cat and canine PrPs using Swiss-Viewer. (Figure 6b,c). In canine PrP, Asp163 was predicted to have a hydrogen bond distance of 2.54 Å with the Met138 (Figure 6b). In cat PrP, Asn166 was predicted to have hydrogen bond distance of 3.16 Å with Met141 (Figure 6c). This result may indicate that the hydrogen bond distance between Asp163 and Met138 of canine PrP is shorter than that between Asn166 and Met141 of cat PrP.

### 3.7. Investigation of the Influence according to Substitution of Cat PrP

**Figure 7:**
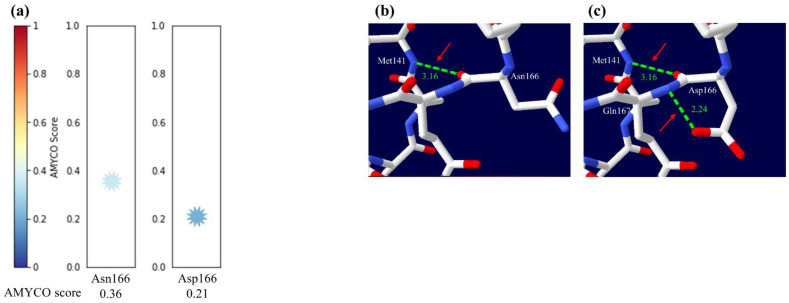
Prediction of the impact on dog-specific amino acid substitution of cat prion protein (PrP). (**a**) Prediction of amyloid propensity according to substitutions to dog-specific amino acids at codon 166 of cat PrP. (**b**) 3D analysis of the Asn166 of cat PrP. Green dotted line indicates a hydrogen bond. The red arrow indicates a hydrogen bond between Asn166 and Met141. (**c**) 3D analysis of the Asp166 of cat PrP. Green dotted lines indicate hydrogen bonds. The red arrows indicate hydrogen bonds between Asp166 and Met141/Gln167.

We estimated the influence on dog-specific amino acid substitution, Asp166 at Asn166 of cat PrP using PolyPhen-2, PANTHER and PROVEAN. The Asp166 was estimated to be “benign” and “neutral” with scores of 0.000 and −1.173 by PolyPhen-2 and PROVEAN, respectively. However, the Asp166 was estimated to be “possibly damaging” with a score of 220 by PANTHER (Table 5). In addition, the cat PrP with Asp166 showed a lower amyloid propensity than that with Asn166 (Figure 7a). Finally, we performed 3D structural analysis to predict a structural change according to the substitution of amino acids at codon 166 of cat PrP (Figure 7b,c). Asn166 was predicted to have a hydrogen bond with Met141 at a 3.16 Å distance (Figure 7b). Remarkably, Asp166 was predicted to have two hydrogen bonds with Met141 at a 3.16 Å distance and Gln167 at a 2.24 Å distance (Figure 7c).

## 4. Discussion

In the present study, we performed amplicon sequencing in 208 domestic cats and found 1 In/Del in the nonapeptide repeat region and 14 novel SNPs, including two nonsynonymous SNPs for the first time (Figure 1 and Figure 2, Table 1). In a previous study, the polymorphisms of cat *PRNP* were mentioned. However, the authors did not provide the information on polymorphisms, including the location, genotype frequencies and base substitution [28]. Although 15 polymorphisms were found in this study, main breeds of cats analyzed in this study is Korean Domestic Shorthair (80.76%) and the numbers of remaining 18 breeds are not enough to represent genetic variations of each local cat breeds. Thus, further investigation in sufficient samples of cats from each local breed is highly desirable in the future. In addition, since polymorphisms shared with other species analyzed in previous studies provide clues to understand a physiology of the cat *PRNP* polymorphisms, further investigation of polymorphisms shared with other species is needed. Furthermore, we found several rare and common variants (Table 1). These results indicate that temporal difference in the occurrence of the variants [47]. We also identified the SNPs, c.-3G>A and c.171C>T showing strong LD with c.797G>A and c.201C>T, respectively. These results denote that there is distinct genetic structure among these SNPs via non-random heredity of the alleles.

Since polymorphisms of the *PRNP* gene can influence PrP structure and susceptibility to prion disease, we evaluated the impact of polymorphisms that cause amino acid variation, including Gly43Glu, p.76_84del PHAGGGWGQ and Glu153Lys using in silico tools. Interestingly, these polymorphisms of cat PrP were predicted to be damaging by PROVEAN, PANTHER and PolyPhen-2. Since several neurodegenerative diseases have been related to the accumulation of amyloidized substrate proteins, we estimated the amyloid propensity of Gly43Glu, p.76_84del PHAGGGWGQ and Glu153Lys. Notably, cat PrP with Lys153 showed decreased amyloid propensity compared to that with Glu153. Glu153Lys is located on helix 1, which is known to be important in the conversion of PrP^C^ to PrP^Sc^ [48]. In addition, a previous study reported that mouse PrP with Glu145 showed a higher conversion rate of PrP^C^ to PrP^Sc^ than that with Lys145 in the ScN2A cell line. Codon 145 in mouse PrP is equivalent to codon 153 in cat PrP [49]. Thus, we estimated the impact of cat PrP on Glu153Lys using 3D structural analysis. Notably, we identified the distributional difference of hydrogen bonds according to Glu153 and Lys153 of cat PrP (Figure 4). In detail, cat PrP with Lys153 has additional hydrogen bonds compared to that with Glu153. Since hydrogen bonds can affect the stability of proteins [50], Glu153Lys may be related to resistance to FSE. However, since our analysis is based on single genetic variation, further analysis based on haplotypes of cat *PRNP* polymorphisms is highly desirable in the future.

Next, to identify structural differences that can affect the difference in resistance to prion disease, we compared the core region of the canine PrP related to resistance to prion diseases with the corresponding region of cat PrP. (Figure 6). The aspartic acid at codon 163 of canine PrP is known as a specific amino acid for the resistance of prion disease, and codon 166 of cat PrP is equivalent to codon 163 of canine PrP. In this region, both codon 163 of canine PrP and codon 166 of cat PrP have one hydrogen bond; however, these codons showed a difference in the length of hydrogen bonds. In detail, Asn166 of cat PrP has a longer hydrogen bond length with Met141 (3.16 Å) than Asp163 of canine PrP with Met 138 (2.54 Å). Since the length of the hydrogen bond is related to strength of the hydrogen bond [51], this result suggests that cats have weaker hydrogen bonds than dogs. Because the weak hydrogen bond is associated with the stability of protein [50] and the stability of PrP influences the conversion of PrP^Sc^ [33,45], these results are remarkable.

Finally, to identify the impact on the substitution of dog-specific amino acids on cat PrP, we evaluated amyloid propensity and performed 3D structural analysis on cat PrP (Figure 7, Table 5). Notably, the substitution of dog-specific amino acids resulted in a decrease in the amyloid propensity of cat PrP (Figure 7a). In addition, cat PrP with Asp166 has additional hydrogen bonds compared to that with Asn166. In a previous study, the canine PrP transgenic mouse with Asp163Asn substitution was shown to be infected with a prion strain, including sheep-BSE [29]. These results suggest that the Asp163 amino acid of canine PrP plays a pivotal role in the resistance of prion disease. Interestingly, we observed that dog specific amino acids in cat PrP affect the amyloid propensity and stability of prion protein. However, since we performed a quite simplified analysis with 3D snapshots of protein, there was limitation, which cannot reflect dynamics of PrP according to alleles of genetic variations. Further investigation based on molecular dynamics is highly desirable in the future [52,53]. Furthermore, these in silico analyses need to be verified using in vivo infection experiments. Thus, the infection study of prion agents, including scrapie strains in cat PrP transgenic mice according to nonsynonymous polymorphisms and dog-specific amino acids is highly desirable in the future.

## 5. Conclusions

In this study, we identified 15 genetic variations, including 14 novel SNPs, and found strong LD and 7 major haplotypes among 15 polymorphisms. Among these polymorphisms, the Gly43Glu, p.72_80delPHAGGGWGQ and Glu153Lys polymorphisms of cat PrP were predicted to be damaging. In addition, Lys153 of cat PrP showed a lower amyloid propensity than Glu153 and was predicted to have one hydrogen bond. However, The Glu153 was predicted to have no hydrogen bond. Furthermore, the hydrogen bond between Asn166 and Met141 of cat PrP was predicted to be longer than that between Asp163 and Met138 of canine PrP. Finally, Asp166 of cat PrP showed a lower amyloid propensity than the Asn166 of cat PrP, which is homologous to Asn163 of canine PrP. Interestingly, Asn166 and Asp166 of cat PrP were predicted to have one and two hydrogen bonds, respectively.

## Figures and Tables

**Table 1:** Genotype and allele frequencies of prion protein gene (*PRNP*) polymorphisms in cats.

Polymorphisms	Genotype Frequency, *n* (%)			Allele Frequency, *n* (%)		HWE
c.-3G>A	GG 205 (98.56)	GA 3 (1.44)	AA 0 (0)	G 413 (99.28)	A 3 (0.72)	0.916
c.128G>A	GG 207 (99.52)	GA 1 (0.48)	AA 0 (0)	G 415 (99.76)	A 1 (0.24)	0.972
c.171C>T	CC 84 (40.38)	CT 64 (30.77)	TT 60 (28.85)	C 232 (55.77)	T 184 (44.23)	0.000
c.201C>T	CC 111 (53.37)	CT 51 (24.52)	TT 46 (22.12)	C 273 (65.63)	T 143 (34.38)	0.000
c.214_240delCCC CACGCCGGCGGA GGCTGGGGTCAG	WT/WT 200 (96.15)	WT/DEL 8 (3.85)	DEL/DEL 0 (0.0)	WT 408 (98.08)	DEL 8 (1.82)	0.777
c.255T>C, G	TT 178 (85.58)	TC 12 (5.77)	CC 0 (0)	T 386 (92.79)	C 12 (2.88)	0.534
		TG 18 (8.65)	GG 0 (0)		G 18 (4.33)	
c.264T>C	TT 156 (75.00)	TC 42 (20.19)	CC 10 (4.81)	T 354 (85.10)	C 62 (19.90)	0.003
c.279C>T	CC 204 (98.08)	CT 4 (1.92)	TT 0 (0)	T 412 (99.04)	C 4 (0.96)	0.888
c.457G>A	GG 165 (79.33)	GA 42 (20.19)	AA 1 (0.48)	G 372 (89.42)	A 44 (10.58)	0.330
c.714C>T	CC 202 (97.12)	CT 6 (2.88)	TT 0 (0)	C 410 (98.56)	T 6 (1.44)	0.832
c.774C>T	CC 163 (78.37)	CT 27 (12.98)	TT 18 (8.65)	C 353 (84.86)	T 63 (15.14)	0.000
c.787C>T	CC 206 (99.04)	CT 2 (0.96)	TT 0 (0)	C 414 (99.52)	T 2 (0.48)	0.944
c.789G>A	GG 197 (94.71)	GA 9 (4.33)	AA 2 (0.96)	G 403 (96.88)	A 13 (3.13)	0.000
c.790C>T	CC 197 (94.71)	CT 11 (5.29)	TT 0 (0)	C 405 (97.36)	T 11 (2.64)	0.695
c.797G>A	GG 205 (98.56)	GA 3 (1.44)	AA 0 (0)	G 413 (99.28)	A 3 (0.72)	0.916

HWE: Hardy-Weinberg Equilibrium.

**Table 2:** Linkage disequilibrium (LD) analysis with r^2^ values among 15 polymorphisms of the prion protein gene (*PRNP*) in cats.

	c.-3G>A	c.128G>A	c.171C>T	c.201C>T	c.214_240del CCCCACGCCGGCGGAGGCTGGGGTCAG	c.255T>C, G	c.264T>C	c.279C>T	c.457G>A	c.714C>T	c.774C>T	c.787C>T	c.789G>A	c.790C>T	c.797G>A
c.-3G>A	-														
c.128G>A	0	-													
c.171C>T	0.009	0.002	-												
c.201C>T	0.004	0.001	0.415	-											
c.214_240del CCCCACGCCGGCGGAGGCTGGGGTCAG	0	0	0.025	0.01	-										
c.255T>C, G	0.001	0	0.008	0	0.252	-									
c.264T>C	0	0.014	0.11	0.092	0.003	0.008	-								
c.279C>T	0	0	0.012	0.005	0	0.001	0	-							
c.457G>A	0.001	0.02	0.001	0	0.002	0.01	0	0.001	-						
c.714C>T	0	0	0.018	0.008	0.014	0	0.003	0	0.003	-					
c.774C>T	0.011	0	0.134	0.093	0.001	0.005	0.003	0.002	0.002	0.003	-				
c.787C>T	0	0	0.006	0.009	0	0	0.001	0	0.041	0	0.002	-			
c.789G>A	0	0.075	0.026	0.017	0.001	0.003	0.001	0	0	0	0	0	-		
c.790C>T	0.024	0	0.001	0	0.001	0.001	0.001	0	0.001	0	0	0.178	0.001	-	
c.797G>A	1.0	0	0.009	0.004	0	0.001	0	0	0.001	0	0.011	0	0	0.024	-

The value of the strong LD (>0.3) is emphasized in bold.

**Table 3:** Haplotype frequency of the 15 *PRNP* polymorphisms in cats.

Haplotype	c.-3G>A	c.128G>A	c.171C>T	c.201C>T	c.214_240del CCCCACGCCGGCGGAGGCTGGGGTCAG	c.255T>C, G	c.264T>C	c.279C>T	c.457G>A	c.714C>T	c.774C>T	c.787C>T	c.789G>A	c.790C>T	c.797G>A	Frequency (*n* = 416)
ht1	G	G	C	T	Wt	T	T	C	G	C	C	C	G	C	G	124 (0.298)
ht2	G	G	T	C	Wt	T	T	C	G	C	C	C	G	C	G	92 (0.220)
ht3	G	G	T	C	Wt	T	T	C	G	C	T	C	G	C	G	47 (0.114)
ht4	G	G	C	C	Wt	T	C	C	G	C	C	C	G	C	G	42 (0.101)
ht5	G	G	T	C	Wt	T	T	C	A	C	C	C	G	C	G	13 (0.031)
ht6	G	G	C	C	Wt	T	T	C	G	C	C	C	A	C	G	9 (0.022)
ht7	G	G	C	C	Wt	T	T	C	G	C	C	C	G	C	G	8 (0.020)
Others	-	-	-	-	-	-	-	-	-	-	-		-	-	-	81 (0.194)

Others contain rare haplotypes with frequency <0.02.

**Table 4:** *In silico* analysis of nonsynonymous polymorphisms of the prion protein gene (*PRNP*) in cats.

Polymorphism	Method	Score	Prediction
c.128G>A (G43E)	PolyPhen-2	1.0	Probably damaging
	PANTHER	Not scored	Invalid substitution *
	PROVEAN	−1.381	
c.214_240delCCC CACGCCGGCGG AGGCTGGGGTC AG (p. p.72_80del PHAGGGWGQ)	PROVEAN	−13.052	Deleterious
c.457G>A (E153K)	PolyPhen-2	0.998	Probably damaging
	PANTHER	361	Possibly damaging
	PROVEAN	−1.629	Neutral

* Mismatch with PANTHER database sequence.

**Table 5:** *In silico* analysis according to substitutions to dog-specific amino acids at codon 166 of the feline prion protein gene (*PRNP*).

Residue	Substitution	Method	Score	Prediction
Asn166	Asp166	Polyphen-2	0.000	Benign
		PANTHER	220	Possibly damaging
		PROVEAN	−1.173	Neutral

## Data Availability

All data generated or analyzed during this study are available from the corresponding author on reasonable request.

## Abbreviations

TSE	Transmissible spongiform encephalopathy
CJD	Creutzfeldt-Jakob disease
BSE	Bovine spongiform encephalopathy
CWD	Chronic wasting disease
TME	Transmissible mink encephalopathy
FSE	Feline spongiform encephalopathy
MDCK	Madin-Darby canine kidney
RML	Rocky Mountain Laboratory
PMCA	Protein misfolding cyclic amplification
PRNP	Prion protein gene
PrP	Prion protein
SNPs	Single nucleotide polymorphisms
LD	Linkage disequilibrium
EDTA	Ethylenediaminetetraacetic acid
HWE	Hardy-Weinberg equilibrium
ORF	Open reading frame
